# An assessment of branching asymmetry of the tracheobronchial tree

**DOI:** 10.1038/s41598-022-14072-6

**Published:** 2022-06-16

**Authors:** Antonio F. Miguel

**Affiliations:** Institute of Earth Sciences and Department of Physics, School of Science and Technology, University of Evora, Rua Romao Ramalho 59, 7000-671 Évora, Portugal

**Keywords:** Respiration, Biomedical engineering

## Abstract

The tracheobronchial tree is commonly seen to have a systematic branching symmetry, despite being known to have an asymmetrical design. Branching asymmetry allows for uniform airflow and provides robustness against the morphogenesis-related size variability. Here, a constructal approach is used to tracheobronchial tree analysis, and a general model based on entropy generation during breathing process is provided, which holds with asymmetric characteristics of the tree, and the change for inhaling and exhaling air. In contrast to traditional models available in the literature, the entropy generation of inspiration and expiration processes is compared for symmetry and asymmetric designs. This approach unravels the fundamental consequences of asymmetric constraint in the process of breathing and provides justification for the tracheobronchial tree having the same number of bifurcation levels as optimized symmetrical trees.

## Introduction

Tree branched flow networks are ubiquitous in Nature because constitute the best solution to the fundamental access-maximization problem of supplying a flow from a central point (source or sink) to a widely distributed points (volume, area, line)^1^. Along a given number of generations of branches, tree-like flow networks display dichotomous branching with a systematic reduction of their size. For maximum flow access subject to volume constraint, the diameter ratio of successive symmetric airway segments is equal to 2^−1/3^ (~ 0.7937) which is known as Hess–Murray’s law^2,3^. In such optimal tree, the successive symmetric airway lengths are also homothetic with the reduction ratio of 2^−1/3^ (~ 0.7937)^3^.

The tree’s branching structure is a key factor in determining its function^1^. As a result, the branching structure of the tracheobronchial tree plays an important role in the resistance and uniform distribution of airflow, as well as in establishing the conditions so that life-giving oxygen diffuses into the blood supply and carbon dioxide is removed. Starting with the trachea, the tracheobronchial tree is also a dichotomous airways network in which the airways become shorter and narrower over 23 generations^2,4^. Measurements conducted on latex rubber cast preparations of the tracheobronchial tree demonstrate asymmetrical airway branches^4^, and consecutive airway segments show reduction size ratios somewhat greater than those obtained from optimality^4^. Only fully symmetric bifurcating tracheobronchial trees have been studied analytically, and the optimal number of bifurcation levels has been determined to be 23^2,5^. These studies, in addition to assuming symmetrical bifurcations, rely solely on the minimization of a total resistance defined as resistances due to airway friction and diffusive transport in the alveolus, and assume a homothetic reduction ratio for sizes of 2^−1/3^. As a result, relevant factors that undoubtedly affect the respiratory process, such as the degree of asymmetry of the bifurcations, homothetic reduction factors greater than 2^−1/3^ that characterize real lungs^4^, and the airway elasticity are not taken into account.

Asymmetries of many types are linked to tree-like flow systems^4–9^. Beyond the geometric asymmetries in the airways^4^, Andrade et al.^9^ found in a groundbreaking work that the flow patterns under laminar flow are dissimilar for a fully symmetric tree network. This occurs after the second level of bifurcation, where the flow distribution is impacted by the Reynolds number^9^ and directionality of the daughter airways^10^ due to airflow inertia. This result has been confirmed by other authors^10,11^, and it has crucial implications for the tracheobronchial tree's development and function. In terms of ventilation, the degree of asymmetry presented by tracheobronchial tree ensures a shorter average transit time than symmetric trees, and it guarantees that all terminal units (acini) of the tree are uniformly supplied with air^11^. In summary, these findings indicate that asymmetry in tree branches is needed for the respiratory tree to function properly. Although the functional importance of asymmetry in the terms described above is well stated, to the best of the author's knowledge, no study has been published on the additional effect of branching asymmetry on airflow design, or on the possible effect it may have on optimality deviations regarding symmetrical bifurcations. Is the uniformity of air supply the only reason why asymmetrical bifurcating trees outperform symmetric trees? Other specific questions must be addressed, which can be summarized as follows: Is it sufficient to evaluate the performance of the respiratory tree by minimizing convective and diffusive resistances? Is there a cost or benefit to having branching asymmetry in terms of efficiency? What role do homothetic reduction factors play in an asymmetry tracheobronchial tree's performance? Do the 23 bifurcation levels that define the asymmetric respiratory tree correspond to the lowest levels of dissipation? Are the levels of dissipation obtained during air inhalation and exhalation the same? Is it appropriate to evaluate the respiratory tree's performance as a quasi-stationary process?

Constructal law is the thermodynamic approach for the performance of thermo-fluid flow systems by generating design and structure^1,12^. In this paper, a constructal approach is given to study the design of the tracheobronchial tree, focusing on the irreversibility generated by the processes taking place in the system. In addition to the terms associated with gas transport due to friction on the walls of the airways and diffusion in the alveoli, which define the existing models available in the literature, our model considers the contribution due to airway elasticity (which results in the introduction of an essential property called tidal volume into the model), as well as the effect of temperature difference between the ambient air and the interior of the body. Furthermore, branching asymmetry degree and the ability to consider homothetic reduction factors other than 2^−1/3^ are taken into account, which is not the case in previous models. This study, among other things, reveals the tracheobronchial tree's compatibility with performance and robustness.

## Entropy generation and constructal view

The constructal law accounts for the generation of shapes of natural systems and emphasizes the basic role that flows play in any thermodynamic process^1,12^. The tracheobronchial tree is an open thermodynamic system that can be analyzed using the quantitative description of irreversibility together with the constructal approach, based on a quantitative calculation of entropy generation. A result of the second law can be written down mathematically as^13^

1 $${\text{S}} = \sum\limits_{{\text{i}}} {\frac{{{\text{dQ}}_{{\text{i}}} }}{{{\text{T}}_{{\text{i}}} }} + \sum\limits_{{\text{j}}} {\frac{{\phi _{{\text{j}}}^{2} {\text{R}}_{{\text{j}}} }}{{{\text{T}}_{{\text{j}}} }} + {\text{s}}_{{\text{int}}} } }$$

where Q represents the heat entering or leaving the system, ϕ is the volumetric flows rate entering or leaving the system, R is the resistance experienced by the flows, T is the temperature, S is the total entropy generation rate, s_int_ is the entropy generation rate within the system, and the first and the second right-hand terms represent the rate heat conveys entropy in or out and entropy generation rate by flows in or out, respectively.

Inhaling air into the tracheobronchial tree to provide oxygen to tissues, and exhaling air to remove carbon dioxide comprise the breathing process. Long inhalations are associated with quiet and restful breathing and have frequency near 0.1 Hz. The related Womersley parameter (defined as a length scale times the square root of the angular frequency to kinematic viscosity ratio) is close to one. This means that the inertia forces of the flow do not outweigh the viscous forces because the time scale of each breath is long enough. As a result, the airflow can be considered quasi-steady.

Here, we consider that the tracheobronchial tree is accessed by both the heat current and fluid streams. Assuming that the heat added to or rejected from the gas is a polytropic process^14,15^, the entropy generation is given by

2 $${\text{S}}_{{{\text{heat}}}} =\uprho \frac{{\upgamma -\upgamma _{{\text{c}}} }}{{(\upgamma - 1)(\upgamma _{{\text{c}}} - 1)}}{\text{r}}_{{\text{g}}}\phi \ln \left( {\frac{{{\text{T}}_{{\text{r}}} }}{{{\text{T}}_{{\text{o}}} }}} \right)$$

here γ is polytropic index (1.35) and γ_c_ is the heat capacity ratio (1.4), r_g_ is the universal gas constant (287 J/kg K), ϕ is the gas volumetric flow rate (9 × 10^−5^–14 × 10^−5^ m^3^/s at rest), ρ is the gas density (1.1 kg/m^3^), T_r_ is the temperature of respiratory tree (310 K), and T is the ambient temperature.

We can think of two zones in the tracheobronchial tree: the conducting zone, which offers a passageway for gas to travel into and out, and the respiratory zone, which comprises structures (alveolar sacs) that are directly involved in gas exchange. The gas moving through these zones is yet another source of irreversibility in the tracheobronchial tree’s activity.

3 $${\text{S}}_{{{\text{flow}}}} = \frac{{\phi ^{2} }}{{{\text{T}}_{{\text{r}}} }}\sum\limits_{{\text{j}}} {{\text{R}}_{{\text{j}}} }$$

According to morphological evidence, the tracheobronchial tree is a hierarchical network of airways with asymmetrical dichotomous branching^3^. Each parent airway gives rise to a bigger daughter airway (major airway) and a smaller daughter airway (minor airway). The ratio of the sizes of the daughter airways, specifically their diameters and lengths, can be written as

4 $$\frac{{{\text{D}}_{{{\text{d}},{\text{minor}}}} }}{{{\text{D}}_{{{\text{d}},{\text{major}}}} }} = \upalpha _{{\text{D}}} \quad 0 < \upalpha _{{\text{D}}} \le 1$$

5 $$\frac{{{\text{L}}_{{{\text{d}},{\text{minor}}}} }}{{{\text{L}}_{{{\text{d}},{\text{major}}}} }} = \upalpha _{{\text{L}}} \quad 0 < \upalpha _{{\text{L}}} \le 1$$

where D is the diameter, L is the length, and the subscripts d, minor and major mean daughter, minor and major airway. In case of a symmetric tree α_D _= α_L _= 1, however the tracheobronchial tree asymmetry matches with α_D_ and α_L_ of 0.76 and 0.83, respectively^5,6,16^.

According to the Hagen–Poiseuille’s law the airways resistance can be defined as^5,13^

6 $${\text{R}}_{{{\text{bran}}}} = \frac{{128\upmu }}{\uppi }\frac{{{\text{L}}_{0} }}{{{\text{D}}_{0}^{4} }}\left( {\frac{{1 - {\text{z}}*^{{{\text{N}} - 1}} }}{{1 - {\text{z}}*}}} \right)$$

with

7 $${\text{z}}* = \frac{{\upalpha _{{\text{L}}} \frac{{{\text{L}}_{{{\text{d}},{\text{major}}}} }}{{{\text{L}}_{0} }}}}{{\left( {1 + \frac{{\upalpha _{{\text{D}}}^{4} }}{{\upalpha _{{\text{L}}} }}} \right)\left( {\frac{{{\text{D}}_{{{\text{d}},{\text{major}}}} }}{{{\text{D}}_{0} }}} \right)^{4} }}$$

where µ is the dynamic viscosity, and D_0_ (~ 0.018 m) and L_0_ (~ 0.12 m) are the diameter and the length of the first airway of the tracheobronchial tree (trachea), respectively. The branching observed in the tracheobronchial tree is consistent with the average reduction factors L_d,major_/L_0_ and D_d,major_/D_0_ between generations of 0.81 and 0.84, respectively^5,6,16^. These length and diameter reduction ratios are not far off from the optimality of 2^−1/3^ (0.794), which characterizes symmetrical trees^2,4^.

The concentration of oxygen/carbon dioxide in the alveoli tissue is different than that within the alveoli. To calculate the associated resistance of 2^N^ alveolar sacs with thickness δ = 10^−4^ d_av_^5,16^ and diameter ${\text{d}}_{{{\text{av}}}} = \frac{{\left( {\upalpha _{{\text{L}}} \frac{{{\text{L}}_{{{\text{d}},{\text{major}}}} }}{{{\text{L}}_{0} }}} \right)^{{{\text{N}} + 1}} }}{{\left( {1 - \upalpha _{{\text{L}}} \frac{{{\text{L}}_{{{\text{d}},{\text{major}}}} }}{{{\text{L}}_{0} }}} \right)}}{\text{L}}_{0}$, an approach can be drawn up assuming a Fickian diffusion

8 $${\text{R}}_{{{\text{diff}}}} = \frac{{{\uprho \delta }{\text{R}}_{{{\text{O}}_{2} \_{\text{CO}}_{2} }} {\text{T}}_{{\text{r}}} }}{{\uppi \Omega_{{{\text{O}}_{2} \_{\text{CO}}_{2} }} {\text{d}}_{{{\text{av}}}}^{2} 2^{{\text{N}}} }} = \frac{{10^{ - 4}\uprho {\text{R}}_{{{\text{O}}_{2} \_{\text{CO}}_{2} }} {\text{T}}_{{\text{r}}} \left( {1 - \upalpha _{{\text{L}}} \frac{{{\text{L}}_{{{\text{d}},{\text{major}}}} }}{{{\text{L}}_{0} }}} \right)}}{{\uppi \Omega_{{{\text{O}}_{2} \_{\text{CO}}_{2} }} {\text{L}}_{0} \left[ {\left( {\upalpha _{{\text{L}}} \frac{{{\text{L}}_{{{\text{d}},{\text{major}}}} }}{{{\text{L}}_{0} }}} \right)^{{{\text{N}} + 1}} } \right]2^{{\text{N}}} }}$$

where ${\text{R}}_{{{\text{O}}_{{2}} \_{\text{CO}}_{{2}} }}$, $\Omega_{{{\text{O}}_{{2}} \_{\text{CO}}_{{2}} }}$ are the gas constant (260 J/kgK, 189 J/kgK), and the diffusivity (2.5 × 10^−9^ m^2^/s, 1.9 × 10^−9^ m^2^/s) for oxygen or carbon dioxide, respectively.

To drive the airflow, the elasticity of muscles and lung tissues (stretching and unfolding fibers) play a key role. During inspiration and expiration processes, the volume of the respiratory tree expands and returns to a volume capacity called Functional Residual Capacity, respectively. The entropy associated to this volume change is given by

9 $${\text{S}}_{{\Delta {\text{V}}}} =\phi \frac{{{\text{B}}_{{{\text{bm}}}} }}{{{\text{T}}_{{\text{r}}} }}\frac{{\Delta {\text{V}}}}{{{\text{V}}_{{\text{f}}} }}\ln \left( {\frac{{{\text{V}}_{{\text{f}}} }}{{{\text{V}}_{{{\text{FRC}}}} }}} \right)$$

with

10 $${\text{V}}_{{{\text{FRC}}}} = \frac{\uppi }{4}{\text{D}}_{0}^{2} {\text{L}}_{0} + \sum\limits_{{{\text{i}} = 1}}^{{\text{N}}} {(1 + \upalpha _{{\text{D}}}^{2} \upalpha _{{\text{L}}} )^{{\text{i}}} \left( {{\text{D}}_{{{\text{d}},{\text{major}}}}^{2} {\text{L}}_{{{\text{d}},{\text{major}}}} } \right)_{{\text{i}}} } = \frac{\uppi }{4}{\text{D}}_{0}^{2} {\text{L}}_{0} \frac{{1 - {\text{w}}*^{{{\text{N}} + 1}} }}{{1 - {\text{w}}*}}$$

and

11 $${\text{w}}* = \left( {1 + \upalpha _{{\text{D}}}^{2} \upalpha _{{\text{L}}} } \right)\left( {\frac{{{\text{D}}_{{{\text{d}},{\text{major}}}} }}{{{\text{D}}_{0} }}} \right)^{2} \frac{{{\text{L}}_{{{\text{d}},{\text{major}}}} }}{{{\text{L}}_{0} }}$$

where B_bm_ is the bulk modulus of the respiratory tree (10^3^–10^4^ Pa)^14^, V_f_ is the volume of the respiratory tree after inspiration (4 × 10^3^–6 × 10^3^ m^3^)^14^, V_FRC_ is the volume of the respiratory tree at Functional Residual Capacity, and ΔV ( = V_f_−V_FRC_) is the tidal volume (45 × 10^−5^–55 × 10^−5^ m^3^)^14^.

According to Eq. (1), combining Eqs. (2), (3), and (9) yield the destruction of useful power and the total entropy generation can be expressed as

12 $$\begin{aligned} {\text{S}} & = {\phi \rho }\frac{{\upgamma -\upgamma _{{\text{c}}} }}{{(\upgamma - 1)(\upgamma _{{\text{c}}} - 1)}}{\text{r}}_{{\text{g}}} \ln \left( {\frac{{{\text{T}}_{{\text{r}}} }}{{{\text{T}}_{{\text{o}}} }}} \right) +\phi \frac{{{\text{B}}_{{{\text{bm}}}} }}{{{\text{T}}_{{\text{r}}} }}\frac{{\Delta {\text{V}}}}{{{\text{V}}_{{\text{f}}} }}\ln \left( {\frac{{{\text{V}}_{{\text{f}}} }}{{{\text{V}}_{{{\text{FRC}}}} }}} \right) \\ & \quad + \;\frac{{\phi ^{2} }}{{{\text{T}}_{{\text{r}}} }}\left\{ {\frac{{128\upmu }}{\uppi }\frac{{{\text{L}}_{0} }}{{{\text{D}}_{0}^{4} }}\left( {\frac{{1 - {\text{z}}*^{{{\text{N}} - 1}} }}{{1 - {\text{z}}*}}} \right) + \frac{{10^{ - 4}\uprho {\text{R}}_{{{\text{O}}_{2} \_{\text{CO}}_{2} }} {\text{T}}_{{\text{r}}} \left( {1 - \upalpha _{{\text{L}}} \frac{{{\text{L}}_{{{\text{d}},{\text{major}}}} }}{{{\text{L}}_{0} }}} \right)}}{{\uppi \Omega_{{{\text{O}}_{2} \_{\text{CO}}_{2} }} {\text{L}}_{0} \left[ {\left( {\upalpha _{{\text{L}}} \frac{{{\text{L}}_{{{\text{d}},{\text{major}}}} }}{{{\text{L}}_{0} }}} \right)^{{{\text{N}} + 1}} } \right]2^{{\text{N}}} }}} \right\} \\ \end{aligned}$$

where z and V_FRC_ are given by Eqs. (7) and (10). According to Eq. (12), the degrees of freedom of the tracheobronchial tree from a design standpoint are D_d,major_/D_0_, L_d,major_/L_0_, α_D_, α_L_, and N.

## Results

The typical values for D_d,major_/D_0_, L_d,major_/L_0_, α_D_, and α_L_, obtained both based on casts of the tracheobronchial tree (asymmetric tree) and optimal reduction size ratios (symmetric tree) are applied in the preceding analysis. Because the values of B_bm_, V_f,_ and ΔV vary over a range of values, a study was conducted to determine the effect of these values on the total entropy S determined by Eq. (12). For both symmetrical and asymmetrical bifurcations, the total entropy generation rate differs by less than 1%. This indicates the respiratory trees’ robustness to the change of these structural parameters. In the following calculations, the mean value of each interval is used, namely B_bm _= 5.5 × 10^3^ Pa, V_f_ = 5 × 10^3^ m^3^, and ΔV = 50 × 10^−5^ m^3^.

**Figure 1:**
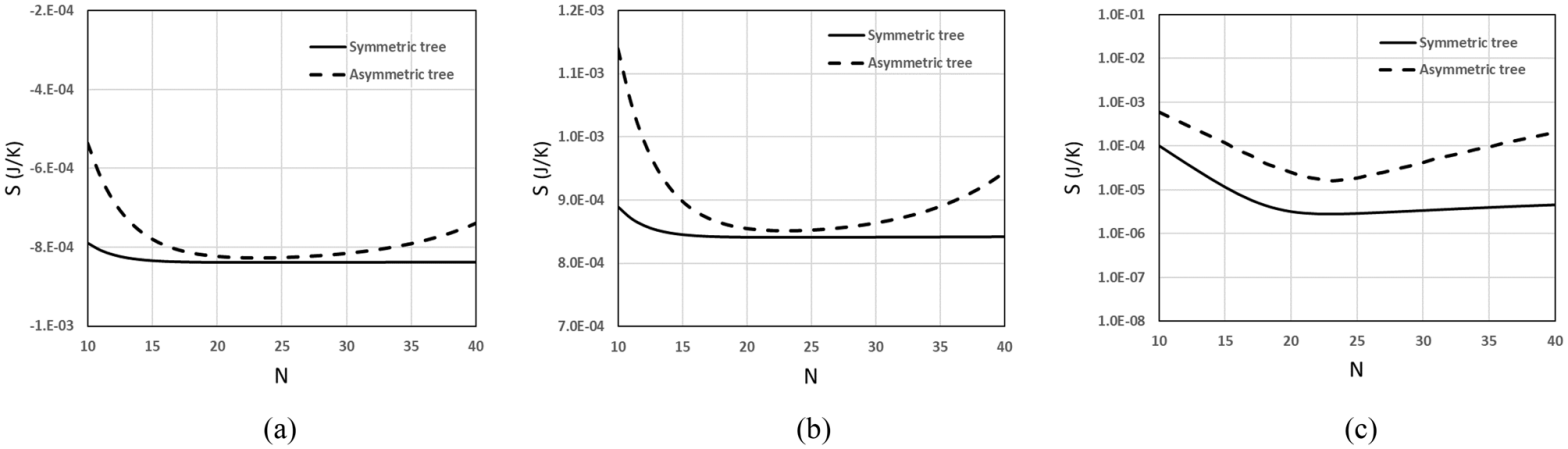
Entropy generation rate vs number of bifurcation levels: (**a**) inspiration (breathing in), (**b**) expiration (breathing out), and (**c**) respiratory cycle (one sequence of inspiration and expiration).

Figure 1 depicts the total entropy generation rate for respiratory trees with symmetrical and asymmetrical bifurcations. This figure reports that the total entropy generation rate by the tree with symmetric branching is less than that generated by the tree with asymmetric branching. Besides, the inspiration process of air may result in a negative S for T_o _< T_r_ (i.e., air flows against a temperature gradient) while the process of exhaling air results in a positive S. However, the global breathing process results in an increase in entropy generation rate. Remarkable is also that curves that relate S to the N present the same minimum value for both inspiration and expiry processes in both symmetric and asymmetric trees. This minimal entropy generation rate occurs for N = 23 (i.e.,23 bifurcation levels), which is consistent with tracheobronchial tree data.

## Discussion

According to Reis et al.^2^ 23 levels of bifurcation correspond to the minimal resistance obtained when the respiratory tree is assumed to be symmetrical, and the size reduction ratio is equal to 2^−1/3^. The tracheobronchial tree lacks branching symmetry and has a size ratio of successive airway segments slightly different from 2^−1/3^, and benefits from this design. A given level of asymmetry ensures that all terminal units of the tree receive the same amount of air, increasing the tree's robustness against the inherent size variability associated with morphogenesis.

The results provided by Eq. (12) show that the entropy generated by the inspiration and expiration processes is extremely unequal, with the value for expiration being about one order of magnitude greater in absolute value. This means that, in addition to serving different purposes, inspiration and expiration have distinct thermodynamic performances. It also shows that for an optimal symmetric tree, 23 levels of bifurcation correspond to the lowest entropy generation rate, which is consistent with values found in the literature by minimizing convective and diffusive resistances. Furthermore, it demonstrates that as long as the asymmetry matches the asymmetry measured in the respiratory tree, whose length reduction ratio is slightly greater than the corresponding optimality recorded in the symmetric tree, the entropy generated by the 23 bifurcation levels is almost identical to that generated by the symmetrical tree. This suggests that the respiratory tree's structure has a degree of asymmetry that allows for the above benefits at the expense of a minor increase in the (lower) entropy generation rate found in symmetrical trees. Our results also show that 23 bifurcation levels are appropriate for both air inspiration (supply of fresh air to the tracheobronchial tree’s extremities) and expiration (carbon dioxide removal from all extremities of tree). As expected, the breathing process increases the entropy generation rate.

In summary, asymmetric tracheobronchial trees with 23 levels of bifurcation provide uniform ventilation and suitable conditions for gas exchange at the tree's terminal units, as well as robustness against the unavoidable size variation caused by morphogenesis, all at a small additional cost specified by the entropy generation rate compared to optimal symmetric trees.
